# “First in Man”: Case Report of Selective C-Reactive Protein Apheresis in a Patient with Acute ST Segment Elevation Myocardial Infarction

**DOI:** 10.1155/2018/4767105

**Published:** 2018-11-06

**Authors:** Wolfgang Ries, Ahmed Sheriff, Franz Heigl, Oliver Zimmermann, Christoph D. Garlichs, Jan Torzewski

**Affiliations:** ^1^Diakonissen Hospital Flensburg, Medical Clinic, Flensburg, Germany; ^2^Charité University Medicine Berlin, Medical Clinic, Berlin, Germany; ^3^Medical Care Center Kempten-Allgäu, Kempten, Germany; ^4^Cardiovascular Center Oberallgäu-Kempten, Kempten, Germany

## Abstract

C-reactive protein (CRP) may be causative in cardiovascular disease. As yet, no specific CRP inhibitor for human application has been described. A 69-year-old male was referred with ST segment elevation myocardial infarction (STEMI). Typical symptoms of chest pain started at 10.00 p.m. The patient was admitted to the hospital at 1.30 a.m. the next day. As ECG showed anterior wall myocardial infarction, the patient was immediately transferred to successful emergency angioplasty/drug-eluting- (DE-) stenting of the subtotally occluded left anterior descending artery. Consecutively, the hemodynamically stable patient was monitored at the chest pain unit. C-reactive protein (CRP) apheresis using the CRP adsorber (PentraSorb® CRP) within CAMI-1 trial was performed 34 h and 58 h after the onset of symptoms. In each apheresis session, 6000 ml plasma was treated via peripheral venous access. Plasma CRP levels decreased from 28.77 mg/l to 12.58 mg/l during the first apheresis session and from 24.17 mg/l to 11.55 mg/l during the second session, respectively. No side effects were observed. This is the first report of selective CRP apheresis in a man. The technology offers multiple opportunities to clarify the immunological/pathogenic role of CRP in health and disease.

## 1. Introduction

For more than two decades, the role of C-reactive protein (CRP) in cardiovascular disease has been controversially and emotionally discussed. Divergent data and opinions have left the scientific community in doubt as to whether CRP is causal in cardiovascular disease or not [1–3]. As interleukin-1*β* (IL-1*β*) induces IL-6, which in turn induces CRP synthesis in the liver, the CANTOS trial has rapidly revitalized the international interest in the matter [4, 5]. IL-1*β* inhibition, however, is an immunological intervention with many potential side effects. Ultimately, specific CRP inhibition in controlled clinical trials may be the only way to prove or disprove a causative role of CRP in cardiovascular disease [3]. Here, we provide the first report of selective CRP apheresis [6] in a man, a CRP-specific technology that removes CRP from the plasma and may finally help to clarify the immunological/pathogenic role of CRP in health and disease.

## 2. Case Presentation

A 69-year-old male was referred to Cardiovascular Center Oberallgäu-Kempten with ST segment elevation myocardial infarction (STEMI). Typical symptoms of chest pain started at 10.00 p.m. The hemodynamically stable patient was admitted to the hospital at 1.30 a.m. the next day. Medical history revealed adenocarcinoma of the medial rectum (pT1, pN0 (0/14), L0, V0, R0, GII, cM0 (UICC I)) with anterior rectum resection in 2014 and complete remission. Furthermore, the patient suffered from chronic kidney disease, stage 3.

**Figure 1:**
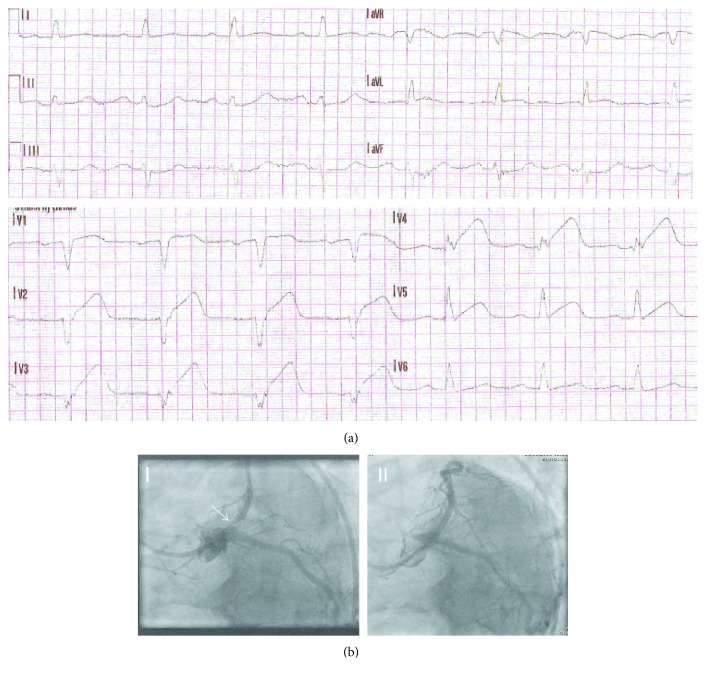
Major patient findings. Emergency ECG at admission (a) documents anterior ST segment elevation in the precordial leads (V1–5) ± the high lateral leads (I and aVL) and reciprocal ST depression in the inferior leads (mainly III and aVF). Spider view of left coronary artery (LAO caudal view LAO 40°, caudal 30°) (b) before (I) and after (II) successful emergency angioplasty/drug-eluting- (DE) stenting. Arrow demonstrates subtotal ostial stenosis with the present thrombus.

**Figure 2:**
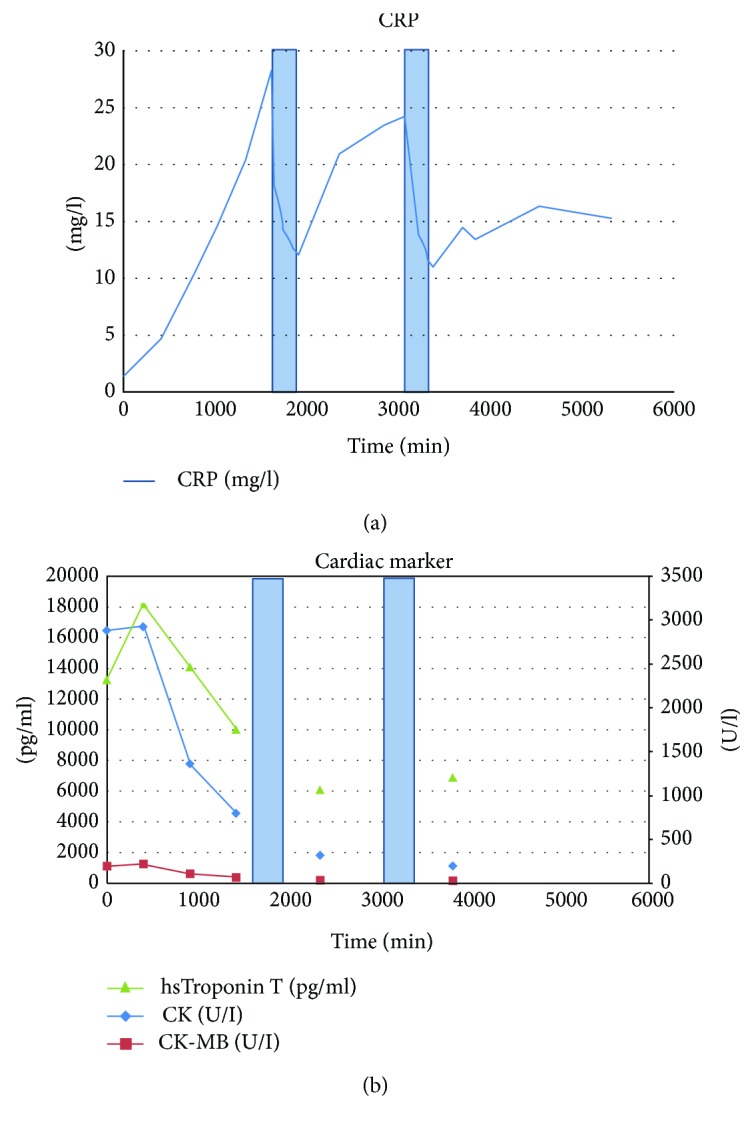
CRP levels and cardiac enzyme progress. CRP levels (a) were normal (normal value 0–5 mg/l) at admission and increased as a result of myocardial necrosis/acute phase reaction as expected. CRP apheresis 1 and 2 (blue columns) 34 h and 58 h after the onset of symptoms (27 h and 51 h after first laboratory results, i.e., zero point in the coordinate system) decreased from 28.77 mg/l to 12.58 mg/l during the first apheresis session and from 24.17 mg/l to 11.55 mg/l during the second session, respectively. CRP apheresis thus efficiently counteracted acute phase CRP elevation. Elevated CK/CK-MB and troponin levels at admission (b) documented acute STEMI. CK levels peaked approximately 14 h after the onset of symptoms and decreased afterwards. *Y*-axis left: pg/ml for hsTroponin T; *Y*-axis right: U/l for CK and CK-MB.

ECG showed anterior wall myocardial infarction (Figure 1(a)). The patient was immediately transferred to the cardiac catheterization laboratory and received successful emergency angioplasty/drug-eluting- (DE) stenting of the subtotally occluded left anterior descending artery (Figure 1(b)). Transthoracic echocardiography showed left ventricular hypertrophy, moderately reduced systolic left ventricular function (LVEF 40%) with anterior, septal, anteroseptal, inferior-apical, and apical hypo- and akinesia. The hemodynamically stable patient was monitored at the chest pain unit. CRP apheresis [7] using the CRP adsorber (PentraSorb® CRP) within C-reactive Protein Apheresis in Acute Myocardial Infarction (CAMI-1) trial [8] was performed 34 h and 58 h after the onset of symptoms. In each apheresis session, 6000 ml plasma was treated via peripheral venous access. Plasma CRP levels declined from 28.77 mg/l to 12.58 mg/l during the first apheresis session and from 24.17 mg/l to 11.55 mg/l during the second session, respectively (Figures 2(a) and 2(b)).  Figure 2 also shows cardiac enzyme progress over 72 h. Elevated creatinine kinase (CK), CK-MB, and troponin levels at admission documented acute STEMI. CRP levels, however, were normal at admission and, as a result of myocardial necrosis, increased with time [9]. CRP apheresis efficiently counteracted acute phase CRP elevation and reduced peak CRP plasma levels.

The patient tolerated apheresis with no clinically relevant symptoms. No side effects were observed, especially signs of infection. The patient was, on his own request, discharged in a good general condition, on day 5 after the onset of symptoms.

## 3. Discussion

Although CRP is known since 1930 [10], not all facets of the molecule's role in the human immune system are yet discovered. Paradoxically, in spite of its widespread clinical use, relatively little is known about CRP's biological functions. The two known CRP functions [11] are as follows: firstly, activation of the classical complement pathway up to C3/C4 via C1q binding and secondly, binding to human immunoglobulin Fc*γ* receptors (mainly Fc*γ*RIIa) after opsonization of biological particles for macrophages [12]. Notably, these functions are also antibody functions. For this reason, it is not unlikely that CRP has been the first antibody-like molecule in the evolution of the mammalian immune system [3]. As CRP functions have been taken over by antibodies with time, CRP may well be an atavism in the human immune system. This hypothesis is underpinned by the complete lack of immunological side effects of selective CRP apheresis in our patient. Nonetheless, the results of CAMI-1 and other carefully designed clinical trials with CRP apheresis have to be awaited. A secondary prevention study in analogy to CANTOS may be conceivable. Also, the role of CRP in stroke [13] or autoimmune disease [14] may be elucidated via selective CRP apheresis.

This is the first report on selective CRP apheresis in a man. CRP apheresis offers multiple opportunities to clarify the immunological and eventually pathogenic role of CRP in health and disease.
